# Comparative Study of Diagnostic Efficacy of Single Phase-Computed Tomography Pulmonary Angiography and Dual Phase-Computed Tomography Pulmonary Angiography in the Diagnosis of Pulmonary Embolism

**DOI:** 10.3389/fcvm.2022.846805

**Published:** 2022-02-25

**Authors:** Xuechun Guan, Qiaoqing Lan, Yi Liang, Honghong Ke, Siqi Chen, Liling Long

**Affiliations:** ^1^Department of Radiology, The First Affiliated Hospital of Guangxi Medical University, Nanning, China; ^2^Department of Pulmonary and Critical Care Medicine, The First Affiliated Hospital of Guangxi Medical University, Nanning, China; ^3^Department of Cardiology, The First Affiliated Hospital of Guangxi Medical University, Nanning, China

**Keywords:** pulmonary embolism, pulmonary hypertension, dual phase-computed tomography pulmonary angiography, single phase-computed tomography pulmonary angiography, false positive, CTPA

## Abstract

**Objective:**

We compared the efficacy of single phase-computed tomography pulmonary angiography (SP-CTPA) and dual phase-computed tomography pulmonary angiography (DP-CTPA) for the diagnosis of pulmonary embolism (PE).

**Methods:**

We recruited 1,019 consecutive patients (359 with PE) who underwent DP-CTPA (phase I: pulmonary artery phase; phase II: aortic phase) for suspected PE between January and October 2021. Phase I of DP-CTPA was used as SP-CTPA, and the final clinical diagnosis (FCD) was used as the gold standard.

**Results:**

Three hundred fifty-two cases of PE were detected by both methods, with the same sensitivity of 98.1% (99.6–99.5%). Using SP-CTPA, 142 cases [13 pulmonary insufficiency artifacts (PIA) and 129 systemic-pulmonary shunt artifacts (S-PSA)] were false-positive with specificity of 78.5% (75.3–81.6%). No false-positive was found with DP-CTPA, with specificity of 100%, positive predictive value of 1, and negative predictive value of 0.990 (Net Reclassification Improvement = 0.215; *P* < 0.05). According to FCD, the positive results of SP-CTPA were divided into PIA, S-PSA, and true-positive (TP_SP−CTPA_) groups, and pairwise comparisons were performed. The bronchiectasis and hemoptysis rate in S-PSA group was higher than that in PIA and TP groups (*P* < 0.001), and the pulmonary hypertension (PH) rate in PIA group was higher than that in S-PSA and TP groups (*P* < 0.001).

**Conclusion:**

The diagnostic efficiency of DP-CTPA for the diagnosis of PE was high. SP-CTPA may misdiagnose PIA (common in patients with PH) and S-PSA (common in patients with bronchiectasis and hemoptysis) as PE.

## Introduction

Pulmonary embolism (PE) is a common and potentially fatal disorder (1–4). PE is one of the major causes of cardiovascular mortality in the United States, accounting for more than 250,000 deaths per year (5). The diagnosis of PE is liable to be missed owing to the non-specific clinical presentation. According to an epidemiological model, in the six European countries with a combined population of 454.4 million in 2004, there were more than 317,000 deaths related to PE, of which sudden fatal PE accounted for 34%; 9% of these cases were not diagnosed before death, and only 7% cases of early deaths were diagnosed before death (6). In a study, a delay (>1.5 h) in the direct communication of an acute PE diagnosis showed a significant correlation with delayed initiation of treatment and higher risk of death within 30 days (7). Thus, timely diagnosis is very important for the clinical treatment of patients with PE. Computed tomography pulmonary angiography (CTPA) is the first choice for the diagnosis of pulmonary embolism (8–10). Currently, both single phase CTPA (SP-CTPA) and dual phase CTPA (DP-CTPA) are used in clinical settings. Compared with DP-CTPA, SP-CTPA is widely used in general hospitals because of its relatively simple operation and low radiation dose (11). DP-CTPA is adopted by some cardiovascular specialist medical centers because the comparative observation of pulmonary circulation and systemic circulation can help avoid false-positive diagnosis of PE, as SP-CTPA only captures the pulmonary artery images quickly (12). The efficacy and applicability of both methods in the diagnosis of PE have not been reported. In this study, we compared the diagnostic efficacy of SP-CTPA and DP-CTPA in the diagnosis of PE, so that the appropriate method can be chosen in clinical work.

## Materials and Methods

### Patient Population and Sample Size Calculation

**Figure 1:**
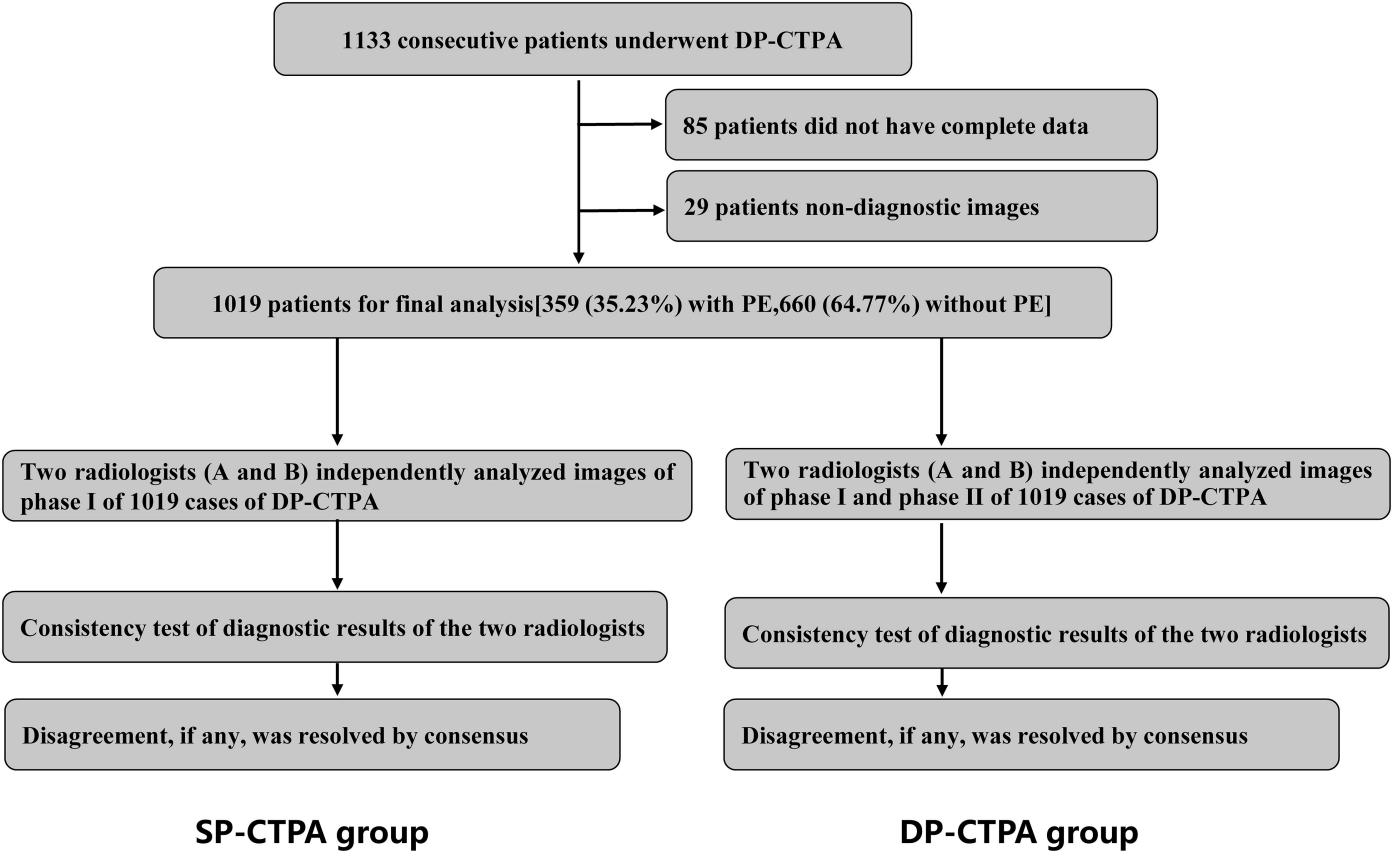
Schematic illustration of the study protocol. We recruited 1,019 consecutive patients with suspected pulmonary embolism **(PE)** who underwent dual phase computed tomography pulmonary angiogram (DP-CTPA, phase I: pulmonary artery phase; phase II: aortic phase) according to the inclusion and exclusion criteria. Phase I DP-CTPA images of all patients were used as the single phase-computed tomography pulmonary angiography **(SP-CTPA)** group. Images of SP-CTPA group were independently analyzed and diagnosed by two experienced radiologists (A and B). Consistency test of diagnostic results of the two radiologists was performed. After an interval of 1 week, the two radiologists analyzed the DP-CTPA group following the same process. The diagnostic efficacy of SP-CTPA and DP-CTPA for the diagnosis of PE was compared using the final clinical diagnosis **(FCD)** as reference.

In the early stage of the study, we assessed the difference between SP-CTPA and DP-CTPA in the diagnosis of PE through a preliminary study. Based on the findings, a sample size of 979 was calculated using the software Power Analysis and Sample Size version 11.0 (PASS 11.0). We recruited 1,133 consecutive patients who underwent DP-CTPA (phase I: pulmonary artery phase; phase II: aortic phase) due to clinical suspicion of PE at our hospital between January 2021 and October 2021. The exclusion criteria were: (1) patients without complete data (*n* = 85);(b) image quality of DP-CTPA did not meet the diagnostic requirements (*n* = 29). Images and data of 1,019 DP-CTPA were included in the final analysis. According to FCD, 359 patients had PE and 660 patients did not have PE (Figure 1). FCD is the final diagnosis obtained after comprehensive examination (including clinical physical examination, laboratory examination, and imaging examination) to identify diseases with similar presentation and after treatment verification. In this study, patients with a FCD of PE were those in whom diseases with similar presentation were excluded based on comprehensive examination and effectiveness of PE treatment (anticoagulation or thrombolysis or pulmonary endarterectomy). Patients without a FCD of PE were those who had other causes of their clinical symptoms, such as pleurisy, acute coronary syndrome, acute aortic syndrome, lung cancer, etc. Patients who did not receive further treatment at our hospital after DP-CTPA or whose etiology was not clear due to lack of complete data were excluded from the study.

### DP-CTPA Scanning and Image Analysis

The main scanning parameters were as follows: tube voltage = 80 kV, automatic tube current modulation (Eff. mAs = 300), pitch = 1.2, rotating speed = 0.28 s, collimation = 128 × 0.6 mm, iterative image reconstruction, reconstruction layer thickness 1 mm, interval 0.6 mm. The intelligent tracking method was used to start the scanning. The monitoring level was the main pulmonary artery with a threshold of 60 HU. After reaching the threshold, the first pulmonary artery phase was scanned in 3S and the second aortic phase was scanned in 6S. A double head power injector was used to inject contrast media. The total amount of contrast agent was 40 mL, the injection speed was 4 mL/s, and 40 mL normal saline was injected at the same injection speed after injection of the contrast agent. All scans were reconstructed and analyzed on the workstation (ADW 4.6, GE Healthcare, Milwaukee, WI, USA).

**Figure 2:**
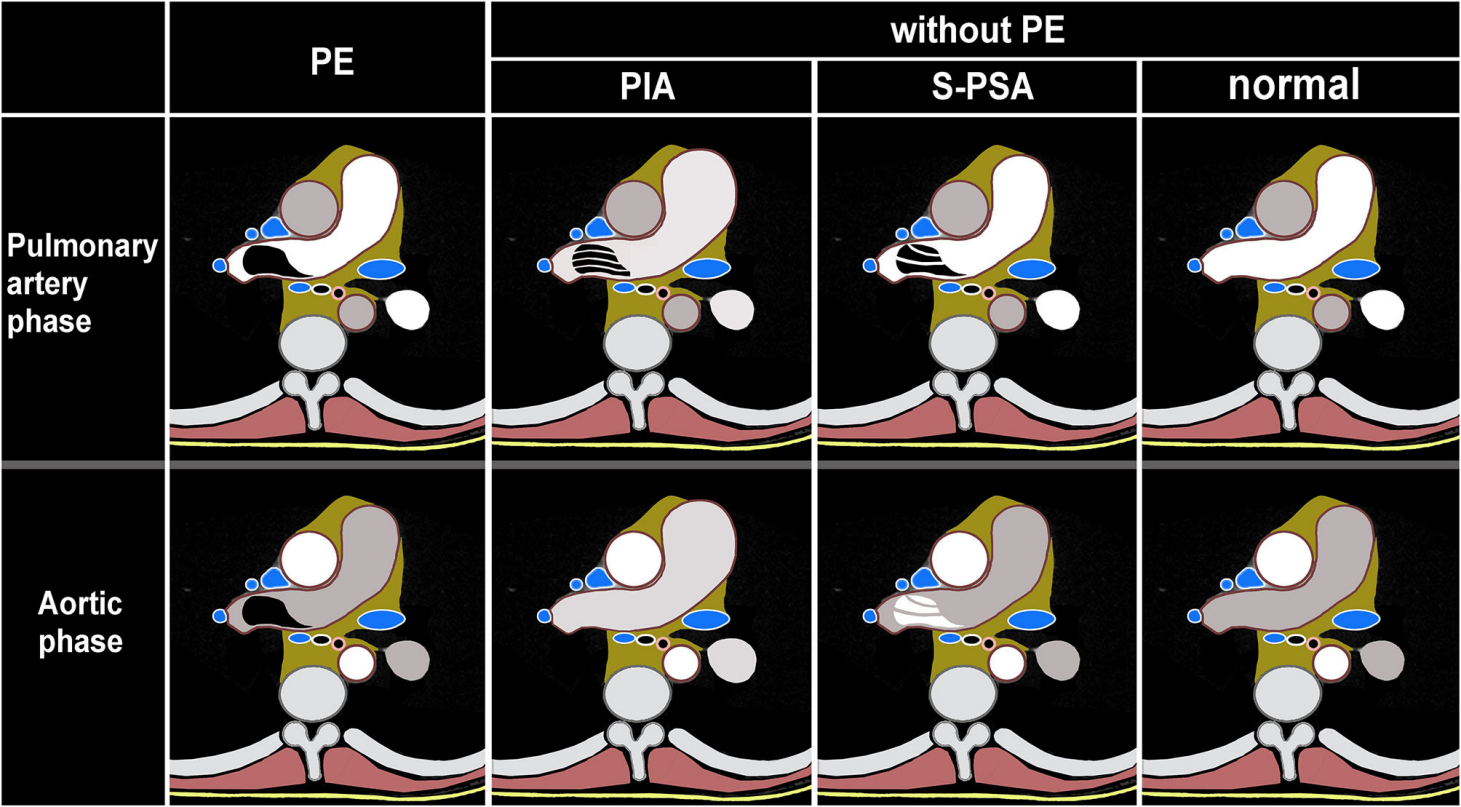
Schematic illustration of dual phase computed tomography pulmonary angiogram (DP-CTPA) diagnosis. PE (pulmonary embolism, Supplementary Video 1) lesion shows low density in pulmonary artery phase and aortic phase. PIA (pulmonary insufficiency artifacts, Supplementary Video 2): The diameter of the main pulmonary artery and the left and right pulmonary arteries increases. During the pulmonary artery phase, the pulmonary arteries exhibit filling defects. During the aortic phase, there is still more contrast media filling in the pulmonary arteries and the filling defect in the pulmonary arteries disappears in the aortic phase. S-PSA (systemic-pulmonary shunt artifacts Supplementary Video 3): In pulmonary artery phase, there is a filling defect or no development of the lumen in the pulmonary artery or its branch. The filling defect or no development of the lumen presents as a high density in the aortic phase. Normal (Supplementary Video 4): There is no filling defect in pulmonary arteries both in pulmonary artery phase and aortic phase.

Images of phase I of all 1,019 cases of DP-CTPA were independently analyzed and diagnosed by two radiologists (A and B) who had more than 5 years of experience in cardiovascular imaging. Consistency of the diagnostic results of the two radiologists were assessed. Disagreement, if any, was resolved by consensus. After an interval of 1 week, the two radiologists analyzed all images (phase I and phase II) of 1,019 cases of DP-CTPA finally included in the study according to the diagnostic approach shown in Figure 2, and made independent diagnoses. Consistency between the diagnostic results of the two radiologists was assessed by Kappa statistic. In case of any disagreement, the issue was resolved by consensus. The diagnostic efficacy of SP-CTPA and DP-CTPA for the diagnosis of pulmonary embolism was compared using the final clinical diagnosis (FCD) as the gold standard.

### Clinical Data

Quantitative assessment of D-dimer levels in plasma was performed by an immunoturbidimetric method (HemosIL D-dimer HS, Instrumentation Laboratory, USA). Cutoff level D-dimer level for defining elevated D-dimer was >450 ng/mL. Hemodynamic and clinical classification of pulmonary hypertension was done according to the 6th World Symposium on Pulmonary Hypertension (13).

### Radiation Dose

An effective radiation dose (ED) was calculated for all patients. The CT scanner recorded the dose-length product (DLP). DLP (mGy·cm) is an indicator of patient dose from CT tube radiation output/exposure. The ED dose was calculated by multiplying DLP by 0.014 mSv/(mGy·cm) (14).

### Statistical Analysis

Data were analyzed using R version 4.1.1 (http://www.r-project.org). Normally-distributed continuous variables were compared using Student's *t*-test, and non-normally distributed continuous variables were compared using the Wilcoxon rank-sum test or Kruskal-Wallis H test. The Chi-squared test or Fisher's exact probability method was used to assess between-group differences with respect to categorical variables. *P*-values < 0.05 were considered indicative of statistical significance.

## Results

A total of 1,019 patients were included in the final analysis, including 660 patients without PE group [59 (49–69) years; male 397, female 263] and 359 with PE group [58 (50–68) years; male 215, female 144]; there were no significant between-group differences with respect to sex or age (Table 1). Patients in the PE group were more prone to chest pain and elevated levels of D-dimer than those in the without PE group (chest pain: 93.59 vs 79.39%; elevated D-dimer levels: 92.76 vs. 49.39%, respectively, *P* < 0.001). Also, more patients experienced hemoptysis in the non-PE group compared with those in the PE group (26.67 vs. 4.18%, *P* < 0.001). In terms of complications, patients in the non-PE group were more likely to be complicated with bronchiectasis (16.21 vs. 0.84%, *P* < 0.001), chronic lung infection (11.82 vs. 2.79%, *P* < 0.001), pulmonary hypertension (7.73 vs. 4.18%, *P* < 0.05), pleural effusion (13.79 vs. 0.84%, *P* < 0.001) and lung cancer (9.97 vs. 2.23%, *P* < 0.001) than those in the PE group.

**Table 1:** Clinical characteristics of patients with PE and without PE.

	**Without PE** ***N*** **= 660**	**With PE** ***N*** **= 359**	***P*** **-value**
Age	59 (49–69)^▴^	58 (50–68)^▴^	0.521
Sex			0.935
Female	263 (39.9%)	144 (40.1%)	
Male	397 (60.1%)	215 (59.9%)	
Chest pain	524 (79.4%)	336 (93.6%)	<0.001^*^
Dyspnea	542 (82.1%)	284 (79.1%)	0.241
Hemoptysis	176 (26.7%)	15 (4.2%)	<0.001^*^
Bronchiectasis	107 (16.2%)	3 (0.8%)	<0.001^*^
Chronic pulmonary infection	78 (11.8%)	10 (2.8%)	<0.001^*^
Pulmonary hypertension	51 (7.7%)	15 (4.2%)	0.028^*^
Pleural effusion	91 (13.8%)	3 (0.84%)	<0.001^*^
Lung cancer	46 (10.0%)	8 (2.2%)	0.001^*^
D-dimer levels increase	326 (49.4%)	333 (92.8%)	<0.001^*^

▴ *Median (IQR)*;

* *P < 0.05; PE, pulmonary Embolism*.

Two experienced doctors evaluated SP-CTPA and DP-CTPA to diagnose patients with a good consistency (Kappa index: 0.919 and 0.916, respectively), and the test results were obtained after discussion. Compared with the FCD, 352 cases of PE were diagnosed with both SP-CTPA and DP-CTPA, with the same sensitivity of 98.1% (99.6–99.5%) (Table 2). One hundred forty-two cases were false positive in SP-CTPA with specificity of 78.5% (75.3–81.6%), positive predictive value of 0.713 and negative predictive value of 0.987. No false positive was found in DP-CTPA, with specificity of 100%, positive predictive value of 1, and negative predictive value of 0.990. The diagnostic ability of DP-CTPA was found to be greater than that of SP-CTPA [Net Reclassification Improvement (NRI) = 0.215; *P* < 0.05].

**Table 2:** Coincidence between single phase-computed tomography pulmonary angiography (SP-CTPA) and dual phase-computed tomography pulmonary angiography (DP-CTPA) in the diagnosis of PE and the FCD.

	**PE (*n* = 359)**	**Without PE** **(*n* = 660)**	**Overall** **1,019**	**Sensitivity (95% CI)**	**Specificity (95% CI)**	**Positive predictive value**	**Negative predictive value**	**NRI**
SP-CTPA				98.1% (96.6–99.5%)	78.5% (75.3–81.6%)	0.713	0.987	0.215^*^
positive	352	142	494					
negative	7	518	525					
DP-CTPA				98.1% (96.6–99.5%)	100.0%	1.000	0.990	
positive	352	0	352					
negative	7	660	667					

*NRI, net reclassification improvement; PE, pulmonary embolism*;

* *z = 11.94, P < 0.05*.

**Figure 3:**
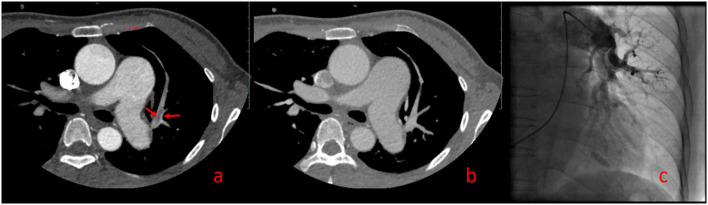
Pulmonary insufficiency artifacts (PIA, Supplementary Video 5). A 39-year-old woman with pulmonary hypertension underwent dual phase-computed tomography pulmonary angiography. A strip filling defect can be seen in the superior lingular branch of left pulmonary artery (SLBLPA), as shown by the arrow in **(a)** (pulmonary artery phase); SLBLPA is well displayed, and there is no filling defect in **(b)** (aortic phase); **(c)** Pulmonary angiography confirmed that there was no PE in SLBLPA.

**Figure 4:**
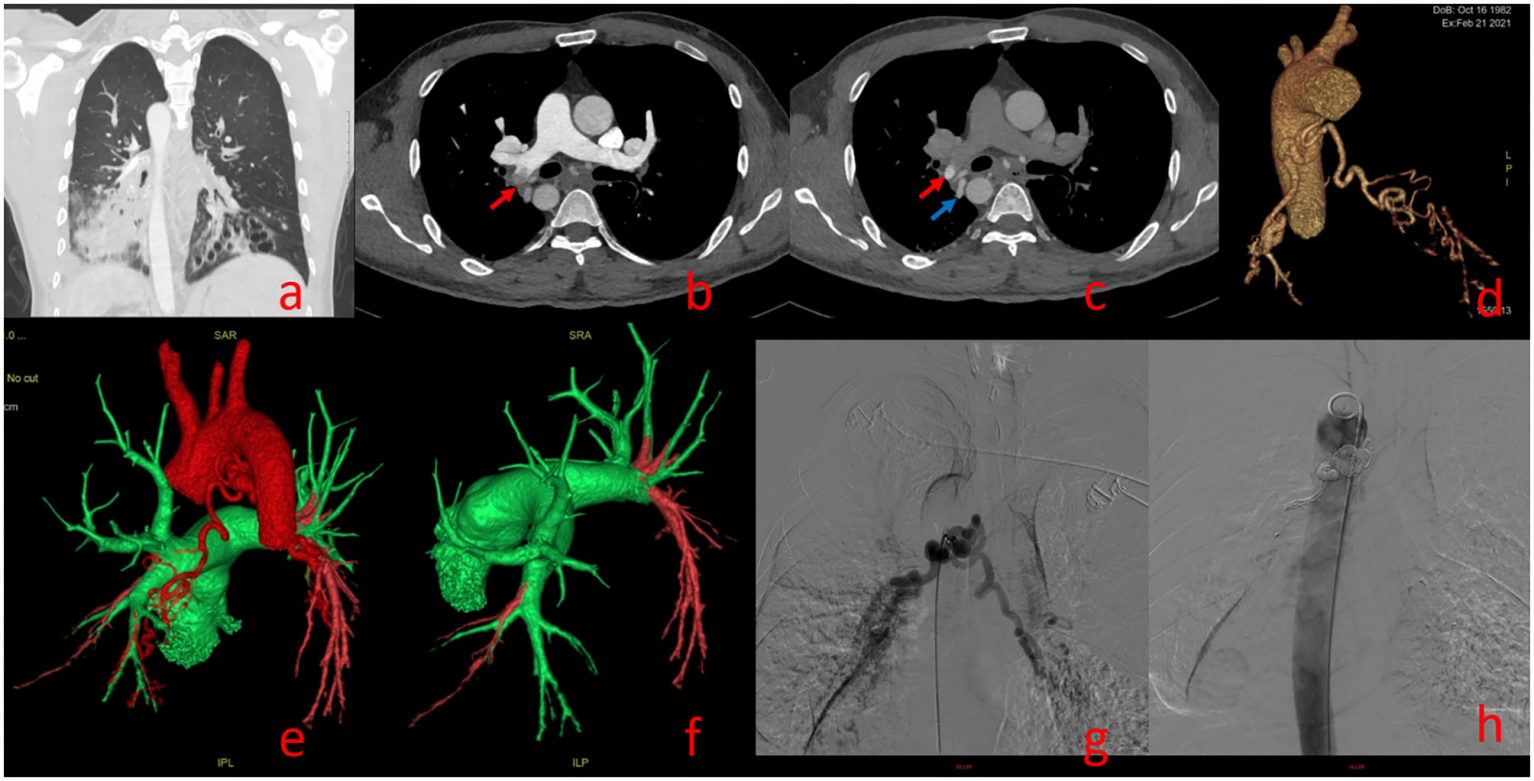
Systemic-Pulmonary Shunt artifacts (Supplementary Video 6). A 39-year-old man with Kartagener syndrome underwent dual phase-computed tomography pulmonary angiography. Bronchiectasis of lower lobes of both lungs in **(a)**. **(b)** (pulmonary artery phase): Filling defect can be seen in the right lower pulmonary artery [red arrow in **(b)**]; **(c)** (aortic phase): the filling defect shown in **(b)** shows high density in **(c)** [red arrow in **(c)**], and its density is similar to that of the adjacent bronchial artery [blue arrow in **(c)**]; Three dimensional reconstruction showing thickening of bronchial artery trunk in **(d)**; **(e,f)** show bronchial-pulmonary artery shunt, Green represents the normal blood flow of pulmonary artery and red represents the normal blood flow of aorta; bronchial-pulmonary artery shunt confirmed by angiography and treated by bronchial artery embolization in **(g,h)**.

One hundred forty-two False Positive PE Cases With Single-Phase CTPA Consisted of 13 Cases of Pulmonary Insufficiency Artifacts (PIA) (Figure 3) and 129 Cases of Systemic-Pulmonary Shunt Artifacts (S-PSA) (Figure 4).

**Figure 5:**
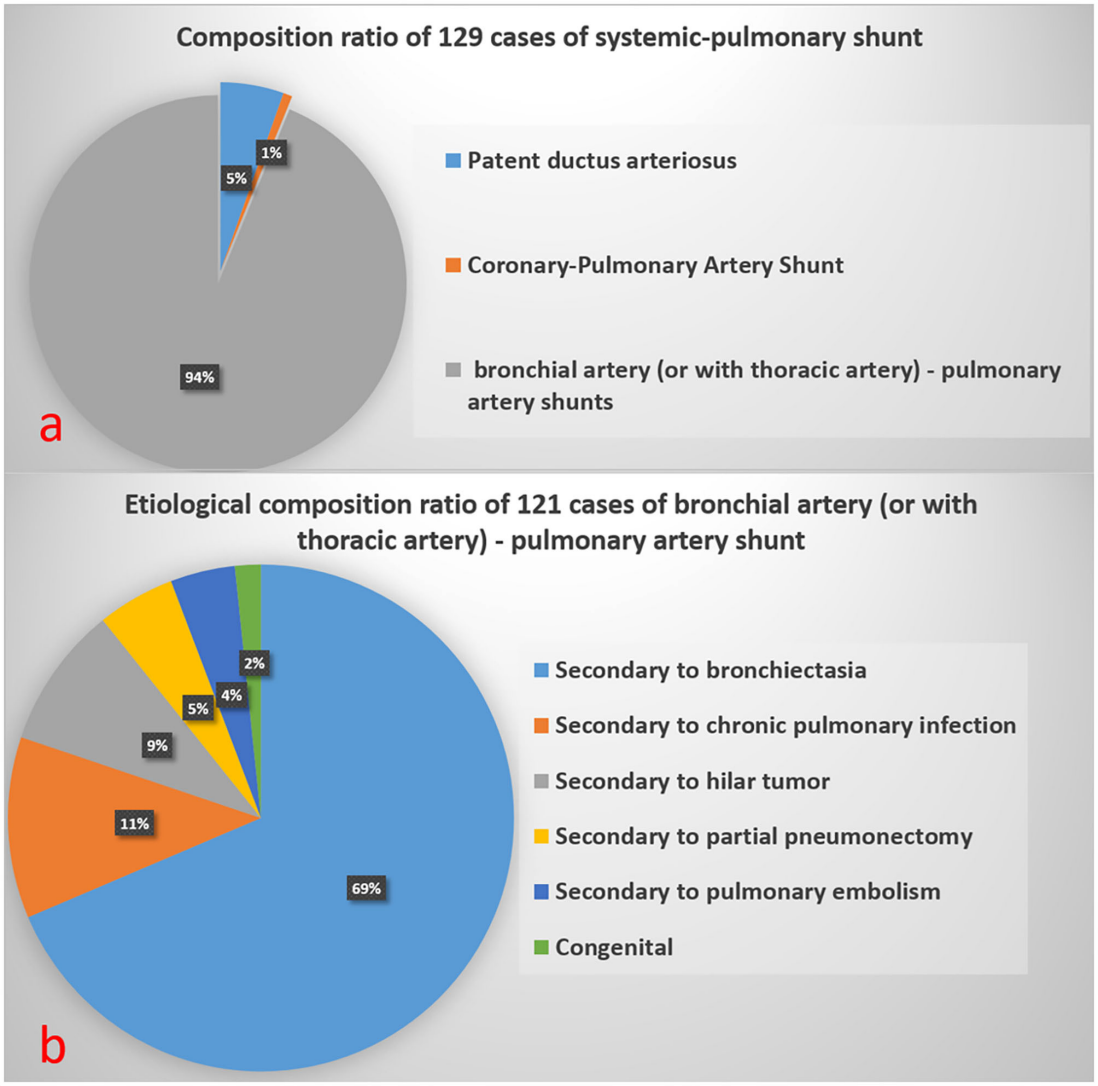
Composition ratio of 129 Systemic-Pulmonary Shunt Artifacts in **(a)**. Etiological composition ratio of 121 cases of bronchial artery (or with thoracic artery)-pulmonary artery shunt in **(b)**.

Figure 5 shows the composition ratio of 129 systemic-pulmonary shunt artifacts (S-PSA).

**Figure 6:**
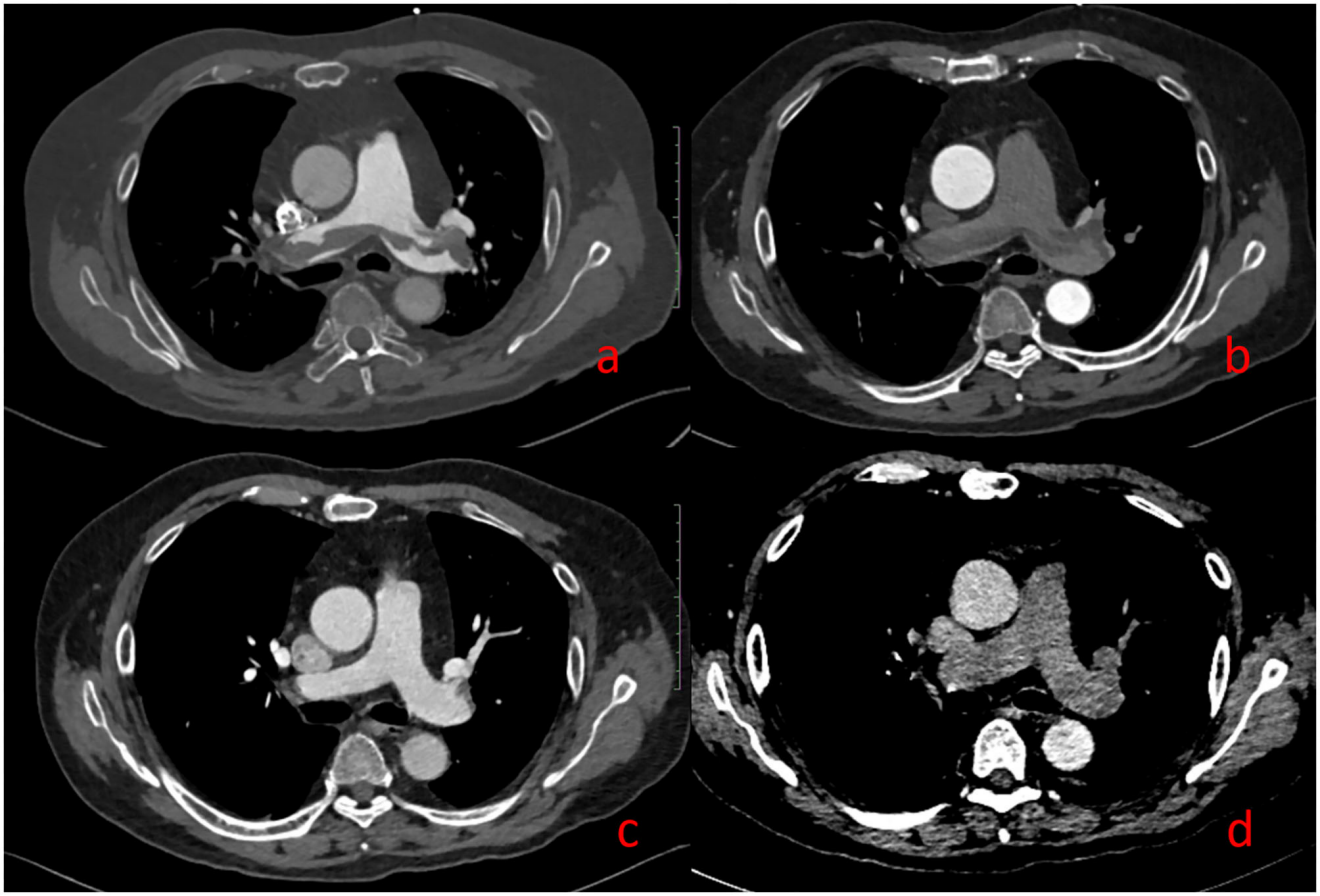
Pulmonary embolism (PE). A 69-year-old man with suspected PE underwent DP-CTPA: Strip filling defects can be seen both in **(a)** (pulmonary artery phase) and **(b)** (aortic phase). After thrombolytic therapy, DP-CTPA was reexamined, and the pulmonary artery filling defect has disappeared in **(c)** (pulmonary artery phase) and **(d)** (aortic phase).

Figure 6 illustrates the image performance of PE in DP-CTPA.

According to FCD, the positive diagnostic results of SP-CTPA were divided into PIA group, S-PSA group, and True-positive (TP SP-CTPA) group. There were significant differences between the three groups with respect to the prevalence of chest pain, dyspnea, hemoptysis, D-dimer increase, bronchiectasis, pulmonary infection, and pulmonary hypertension (Table 3). Specifically, there were significant differences in the prevalence of chest pain and pulmonary hypertension between PIA and TP SP-CTPA groups. With respect to hemoptysis, bronchiectasis and pulmonary hypertension, there were significant differences between the PIA and S-PSA groups. In addition, there were significant differences between S-PSA and TP SP-CTPA groups with respect to chest pain, dyspnea, D-dimer increase, bronchiectasis, and pulmonary infection.

**Table 3:** Comparison of basic data of PIA, S-PSA and TP _SP−CTPA_ groups.

	**PIA (*n* = 13)**	**S-PSA (*n* = 129)**	**TP_**SP−CTPA**_ (*n* = 352)**	***P*** **-value**
Age	60 (47–71)^▴^	59 (45–67)^▴^	58 (50–68)^▴^	0.815
Gender				0.363
Female	3 (23.1%)	44 (34.1%)	138 (39.2%)	
Male	10 (76.9%)	85 (65.9%)	214 (60.8%)	
Chest pain				**<0.001** ^**AC**^
No	4 (30.8%)	60 (46.5%)	23 (6.5%)	
Yes	9 (69.2%)	69 (53.5%)	329 (93.5%)	
Dyspnea				**0.003** ^**C**^
No	4 (30.8%)	12 (9.3%)	75 (21.3%)	
Yes	9 (69.2%)	117 (90.7%)	277 (78.7%)	
Hemoptysis				**<0.001** ^**BC**^
No	13 (100.0%)	14 (10.9%)	337 (95.7%)	
Yes	0 (0.0%)	115 (89.1%)	15 (4.3%)	
D-dimer increase				**<0.001** ^**C**^
No	3 (23.1%)	68 (52.7%)	23 (6.5%)	
Yes	10 (76.9%)	61 (47.3%)	329 (93.5%)	
Bronchiectasia				**<0.001** ^**BC**^
No	13 (100.0%)	46 (35.7%)	349 (99.1%)	
Yes	0 (0.0%)	83 (64.3%)	3 (0.9%)	
Pulmonary infection				**0.003** ^**C**^
No	13 (100.0%)	115 (89.1%)	342 (97.2%)	
Yes	0 (0.0%)	14 (10.9%)	10 (2.8%)	
Pulmonary hypertension				**<0.001** ^**AB**^
No	0 (0.0%)	124 (96.1%)	341 (96.9%)	
Yes	13 (100.0%)	5 (3.9%)	11 (3.1%)	

▴ *Median (IQR). The P-value is the overall statistical test among the three groups. A indicates significant difference between PIA and TP groups; B indicates significant difference between PIA and S-PSA groups; C indicates significant difference between TP and S-PSA groups. PIA, pulmonary insufficiency artifacts; S-PSA, systemic-pulmonary shunt artifacts; TPSP-CTPA, true positive*.

The right heart catheterization parameters of patients with pulmonary hypertension in the PIA group were compared with those in the true negative (TN SP-CTPA) group of SP-CTPA (Table 4). There were significant differences between the PIA and TN groups with respect to pulmonary arterial wedge pressure (PAWP), pulmonary vascular resistance (PVR), and cardiac index (CI) (*P* < 0.05). Patients with pulmonary hypertension in the PIA group had higher PAWP, PVR, and lower CI; there was no significant difference between the two groups with respect to mean pulmonary arterial pressure (mPAP).

**Table 4:** Comparison of RHCP in patients with PH between PIA and TN _SP−CTPA_ groups.

**RHCP**	**PIA (*n* = 13)**	**TN_**SP−CTPA**_ (*n* = 33)**	***P*** **-value**
mPAP (mmHg)^▴^	40 (37–53)	40 (35–53)	0.464
PAWP (mmHg)^Δ^	13.2 ± 4.9	7.6 ± 4.4	0.002
PVR (WU)^▴^	11.4 (10.8–12.3)	6.0 (3.6–10)	0.001
CI (L/min*m^2^)^▴^	1.8 (1.7–2.0)	3.6 (2.9–5.0)	<0.001

▴ *Median (IQR)*;

Δ *Mean ± Sd; RHCP: right heart catheterization parameters; PH, pulmonary hypertension; TNSP-CTPA, Taking FCD as the gold standard, the result of SP-CTPA in the diagnosis of PE was true positive (TP); PIA, Taking FCD as the gold standard, the result of SP CTPA in the diagnosis of PE was false positive caused by pulmonary insufficiency artifacts (PIA); mPAP, mean pulmonary arterial pressure; PAWP, pulmonary arterial wedge pressure; PVR, pulmonary vascular resistance; WU, Wood Units; CI, cardiac index*.

### Radiation Dose

The median ED (mSV) of 1,019 cases of DP-CTPA was 2.0 (interquartile range: 1.8–2.1).

## Discussion

In this study, we found that DP-CTPA offered notable advantages over SP-CTPA in the diagnosis of PE. Three hundred fifty-two cases of PE were detected by both methods, with the same sensitivity. However, use of SP-CTPA led to 142 false positives (13 PIA and 129 S-PSA); therefore, its specificity and positive predictive value were lower than those of DP-CTPA (Table 2). SP-CTPA was unable to distinguish between PE, PIA, and S-PSA. In various guidelines for pulmonary embolism and pulmonary hypertension, CTPA results are an important basis for diagnosis, classification and formulation of diagnosis and treatment strategies (1, 2, 10, 13, 15–18). False-positive diagnosis of PE by SP-CTPA will mislead clinical diagnosis and treatment, and even have serious consequences.

In this study, with FCD as the reference, 13 cases of PIA were falsely diagnosed as PE by SP-CTPA, and 13 cases of PIA were complicated with pulmonary hypertension. By comparing the right heart catheterization parameters of patients with pulmonary hypertension in PIA group and TN SP-CTPA group, we found that patients in the PIA group did not show higher mPAP, but showed higher PAWP, PVR, and lower CI value (Table 4), which is the characteristic of decompensated pulmonary hypertension. We speculate that such patients have increased PVR, decreased cardiac output, prolonged filling time of pulmonary artery and its branches, and partial branches of pulmonary artery cannot be well-filled during SP-CTPA trigger scanning, resulting in PIA (Figure 3a). The exact mechanism of PIA and its significance needs to be confirmed in larger studies. If such patients are misdiagnosed as PE by SP-CTPA, it will lead to the wrong classification of the etiology of PH into the fourth category of PH, which will interfere with the treatment protocols (13, 15, 18). DP-CTPA can distinguish PE and PIA by comparing pulmonary artery phase (phase I) and aortic phase (phase II). PE shows low density (Figures 6a,b) in phase I and phase II. The imaging characteristics of PIA in DP-CTPA are as follows: During the pulmonary artery phase, the diameter of the main pulmonary artery and the left and right pulmonary arteries increases, the inner diameter of the right ventricle increases, and pulmonary arteries or the small branches or branches with large turning points of the pulmonary artery (such as the medial branch of the middle lobe of the right lung and the lingual branch of the upper lobe of the left lung) exhibit filling defects (Figures 2, 3a). During the aortic phase, the filling time of the pulmonary artery is prolonged, which shows that there is still more contrast media filling in the pulmonary artery and its branches in the aortic phase (under normal pulmonary circulation, the pulmonary arteries show contrast agent outflow in the aortic phase), and the filling defect in the pulmonary artery branch disappears in the aortic phase (Figures 2, 3b).

In this study, with FCD as the reference, 129 cases of S-PSA were falsely diagnosed as PE by SP-CTPA. The main blood supply vessels were bronchial artery and/or thoracic artery pulmonary artery shunt, accounting for 94% of all S-PSA (Figure 5a). In this study, the main cause of bronchial artery and/or thoracic artery pulmonary artery fistula was secondary to bronchiectasis, and chronic pulmonary infection was also a common clinical cause. At the bronchial and pulmonary lobular levels, there are many potential anastomoses between the pulmonary artery and the bronchial artery system. Bronchiectasis, chronic lung infection, tumor, and local lung tissue injury can damage local pulmonary circulation, lead to hypoxia, stimulate the release of vascular growth factor, open the anastomotic branch between bronchial artery and pulmonary artery, leading to the expansion and proliferation of bronchial artery and/or thoracic artery, and even bronchial artery and/or thoracic artery pulmonary artery shunt. When exposed to systemic pressure, these dilated and proliferating blood vessels are prone to rupture causing hemoptysis (19). In previous studies on treating massive hemoptysis secondary to infection by embolization of bronchopulmonary artery, the phenomenon of bronchial pulmonary artery shunt was observed (20). In this study, 89.1% of S-PSA patients were complicated with hemoptysis. During SP-CTPA examination, the contrast agent mainly exists in the pulmonary artery and its branches. There is no contrast agent or only a small amount of contrast agent in the aorta. There is a large difference in blood flow density between the aorta and the pulmonary artery. When the patient has systemic-pulmonary shunts, the low-density blood flow from the systemic circulation can appear as filling defect (S-PSA) in the pulmonary artery and its branches. Some patients with systemic-pulmonary shunts have chest pain, shortness of breath, and elevated D-dimer level, so it is usually difficult to distinguish them from PE based on clinical symptoms and laboratory tests. Hemoptysis in patients with systemic-pulmonary shunts was controlled by drugs, interventional embolization of corresponding systemic blood supply arteries, or surgical resection of the corresponding pulmonary lobes. If patients with systemic-pulmonary shunts undergo SP-CTPA, they will be misdiagnosed as PE and administered thrombolytic or anticoagulant therapy, which will lead to massive hemoptysis. DP-CTPA can accurately identify S-PSA by comparing pulmonary artery phase (phase I) with aortic phase (phase II). The imaging features of S-PSA in DP-CTPA are as follows: In pulmonary artery phase (phase I), there is a filling defect or no development of the lumen in the pulmonary artery or its branch lumen (Figures 2, 4b). The filling defect or no development of the lumen presents a high density in the aortic phase (phase II) (Figures 2, 4c), which is much higher than the lumen density of the adjacent normal pulmonary artery (the normal pulmonary artery presents a contrast agent outflow state—low density in the aortic phase). Three-dimensional reconstruction shows dilated bronchial artery or thoracic artery near S-PSA (vessel diameter >2 mm or diameter >30% of the other normal bronchial artery or thoracic artery are dilated). DP-CTPA can accurately identify S-PSA to avoid misdiagnosis as PE. In addition, its phase II is equivalent to aortic CT angiography. It can display important information such as the position, quantity, opening direction and opening diameter of blood supply vessels, to provide support for further treatment (21–24). This simplifies the process and avoids the risks caused by misdiagnosis and repeated inspection. A previous case report described a patient with bronchial artery pulmonary artery fistula secondary to bronchiectasis in whom a false positive diagnosis of PE was made based on CTPA (12). We found that bronchial artery pulmonary artery fistula is not a rare phenomenon, which may be related to more patients with bronchiectasis in this region.

For a long time, improving the scanning scheme and reducing the radiation dose and use of contrast agent in CTPA has been a research focus. These studies are based on SP-CTPA scanning and have achieved great success (9, 11, 25–27). In this study, the scanning scheme was designed based on the above research results to control the total radiation dose of DP-CTPA at an acceptable level. The median ED (mSV) of 1,019 cases of DP-CTPA was 2.0 (1.8–2.1). However, compared with SP-CTPA, which also uses the same low radiation dose technologies such as high pitch and iterative reconstruction, the radiation dose of DP-CTPA is twice as high. In addition, because DP-CTPA needs to well-capture the pulmonary artery phase and aortic phase successively in a single examination, its operation is much more complex than SP-CTPA in which only the pulmonary artery phase is captured. In addition, the requirements for operators and CT equipment for DP-CTPA are relatively high.

On further analysis of our results, 352 cases of PE were detected by both methods, with the same sensitivity of 98.1% (99.6–99.5%) (Table 2). There were 142 false-positive diagnoses of PE by SP-CTPA with a specificity of 78.5% (75.3–81.6%), positive predictive value (PPV) of 0.713, but a high negative predictive value (NPV) of 0.987. On the other hand, no false positives were found with DP-CTPA (specificity = 100%, PPV = 1, and NPV = 0.990). Based on the above data and the advantages and disadvantages of the two methods, we believe that the selective use of DP-CTPA and SP-CTPA in clinical work may offer a distinct advantage. For patients with suspected PE who have other concomitant diseases that are liable to lead to PIA or S-PSA, DP-CTPA should be performed to avoid misdiagnosis. SP-CTPA should be preferred for other patients in order to reduce radiation exposure.

### Study Limitations

Some limitations of this study should be considered while interpreting the results. First, this was a single-center retrospective study. Moreover, the center is a regional national respiratory center; therefore, more patients with bronchiectasis are admitted here compared with other medical centers at the same level in the same region, which may lead to lack of representativeness.

In conclusion, SP-CTPA can misdiagnose PIA (mostly in patients with pulmonary hypertension) and S-PSA (mostly in patients with bronchiectasis and hemoptysis) as PE, causing trouble for clinical diagnosis and treatment; DP-CTPA can effectively distinguish PE, PIA and S-PSA, and its diagnostic efficiency is better than SP-CTPA.

## Data Availability Statement

The original contributions presented in the study are included in the article/Supplementary Material, further inquiries can be directed to the corresponding author/s.

## Ethics Statement

Medical ethical approval was waived as it concerned retrospective analysis of anonymized patient data.

## Author Contributions

XG did the screening of papers and drafted the article. LL is the guarantor. All authors participated in design, aim, contributed substantially to, and approved the final version of the article.

## Funding

This work was supported by the Research Project of Guangxi Health Committee (Z20180916) and Natural Science Foundation of Guangxi (GUIKEAB20238016).

## Conflict of Interest

The authors declare that the research was conducted in the absence of any commercial or financial relationships that could be construed as a potential conflict of interest.

## Publisher's Note

All claims expressed in this article are solely those of the authors and do not necessarily represent those of their affiliated organizations, or those of the publisher, the editors and the reviewers. Any product that may be evaluated in this article, or claim that may be made by its manufacturer, is not guaranteed or endorsed by the publisher.
